# HIV/AIDS-Related Knowledge and Attitudes Among Chinese College Students and Associated Factors: A Cross-Sectional Study

**DOI:** 10.3389/fpubh.2021.804626

**Published:** 2022-01-12

**Authors:** Ling Zhang, Hang Yu, Hong Luo, Wenlong Rong, Xianxin Meng, Xiaoan Du, Xiaodong Tan

**Affiliations:** ^1^School of Public Health, Wuhan University, Wuhan, China; ^2^Youth League Committee, North China University of Water Resources and Electric Power, Zhengzhou, China

**Keywords:** HIV/AIDS, HIV knowledge, student, attitudes, adolescent

## Abstract

In recent years, adolescent has become one of the high-risk groups for HIV. Meanwhile, good HIV awareness and positive attitude are essential for HIV prevention. This study aims to evaluate the extent to which college students understand HIV and their attitudes toward HIV-infected patients, as well as the correlative factors. The data used in this study came from a cross-sectional survey. An anonymous online questionnaire was used to investigate the demographic characteristics, HIV/AIDS-related knowledge, and attitudes toward HIV-infected patients of 17,678 students from a university in Henan. Descriptive statistics, Chi-square test and logistic regression were used to analyze differences and connections between variables in SPSS version 25.0. Participants' HIV/AIDS-related knowledge awareness rate was 80.8%. Levels of students' HIV/AIDS-related knowledge correlated with their gender, nationality, marital status, and their grade (*p* < 0.01). Female students [OR = 0.757, 95% CI (0.699–0.820)] and minority students (OR = 0.717, 95% CI (0.619–0.832)] had insufficient HIV health education knowledge. Meanwhile, male students (OR = 0.845, 95% CI (0.773–0.924)], and students with good HIV knowledge (OR unaware-ness/awareness = 2.385, 95% CI (2.111–2.694)] were more likely to hold a positive attitude toward HIV-infected patients. The relevant education departments should strengthen and promote the education of AIDS transmission and prevention. Many college students still hold negative attitude toward HIV-infected patients. The government should further make efforts to eliminate social discrimination in HIV-infected patients and lead people to approach HIV-infected patients fairly.

## Introduction

AIDS (Acquired Immunodeficiency Syndrome) is an infectious disease caused by HIV (Human Immunodeficiency Virus). The emergence of HIV has brought new challenges to the public health of countries around the world, especially developing countries (1, 2). According to a report by the World Health Organization, in 2020 alone, 410,000 young people aged from 10 to 24 years were newly infected with HIV, of whom 150,000 were adolescents between the ages of 10 and 19 (3).

AIDS was first discovered and reported in China in 1985. In order to better respond to HIV/AIDS, Chinese government has successively promulgated a number of related policies, such as the “Four Frees and One Care,” which have played a very positive role in the prevention and control of HIV in China (4). Although China attaches great importance to HIV health education for young students, related policies have declined after 2006 (5). The awareness rate of HIV health education knowledge among young students (69.22–81.24%) is generally lower than the national standard (90%) [14], especially the misunderstanding of the transmission mode of HIV (6, 7).

China is the most populous country in the world, there are approximately 230 million young people in the 10–24 years age group (8). Due to the gradual liberalization of sexual concepts, the incidence of premarital sex among college students has continued to increase (9), yet sex education has not yet been fully popularized in Chinese families and schools (10). Unprotected sex is one of the high-risk factors for AIDS, especially in low-income countries (11, 12). The lack of sexual knowledge among college students has led to an increase in the rate of HIV infection among college students, which is why college students have gradually become the key monitoring population for AIDS prevention and control in recent years. According to the Chinese Center for Disease Control and Prevention (CDC), the recent increase in the number of HIV infections among college students has increased by 30–50% per year (13). In recent years, more than 95% of newly diagnosed HIV-infected persons in China have been infected through sexual behavior, among which about 70% are heterosexual transmission (14), but the main mode of transmission among young students is male homosexual behavior. The incidence characteristics of AIDS in Henan Province are the same as those of the whole country, with a higher proportion of same-sex transmission among young students aged 15–24 years (15).

Insufficient knowledge about HIV makes people's misunderstanding of AIDS groups continue to deepen, and even affects a person's basic beliefs and leads to discriminatory attitude. Negative attitude makes people less willing to know more about HIV/AIDS. This discrimination has become one of the obstacles to the elimination of HIV discrimination (16). In addition, some news about HIV-infected patients maliciously spreading HIV in society also makes people more resistant to HIV groups. Studies have proved that good education can effectively enhance people's awareness of HIV health education knowledge (17), and good HIV awareness can effectively reduce people's high-risk behaviors and improve people's discrimination against HIV-infected patients (6, 18).

### Significance and Objectives of the Study

This article takes a university in Henan Province as an example to study the awareness of HIV health education knowledge of Chinese college students and their attitudes to live with HIV-positive roommates. In order to discuss the current status and problems of sex education among college students, and explore the influencing factors that affect the level of cognition and attitudes to live with HIV-positive roommates. The final research results can provide references for China's future HIV health education, and at the same time make the popularization of sex education in China more targeted.

## Methods

### Data Sources

This cross-sectional study was carried out in a university in Henan Province in November 2020. After seeking the informed consent of the interviewed students, we collected data in the form of an anonymous online questionnaire. A total of 18,238 questionnaires were distributed to undergraduates in the school, 18,179 were recovered, with a response rate of 99.7%. After excluding the questionnaires with missing values, a total of 17,678 valid questionnaires were included in our analysis. The questionnaire effective response rate was 97.2%. The ethics committee of Wuhan University approved this study (Approval ID:2021YF0047). All informed consents were obtained.

### Survey Design

The HIV health education knowledge questionnaire used in the data collection of this study was adapted from the “adolescent awareness rate” in “Chinese AIDS Sentinel Testing Implementation Plan” and consisted of three sections. The overall reliability and validity test passed, but the load of the entry one factor is too small, so we deleted the first question.

The first section collected the sociological demographic characteristics of the participants including grade, gender, nationality, marital status, monthly living fee level.

The second section used a nine-question questionnaire to assess participants' level of HIV health education knowledge. The score calculation method was: each correct answer in each block of HIV health education knowledge counted for one point. The overall score was the total number of correct responses (ranged from 0 to 8 points). According to the “adolescent awareness rate,” a score of 6 or more was considered to be up to the standard for HIV health education knowledge.

The third section assessed the attitudes of participants toward HIV-infected patients.

### Data Analysis

Data analysis was carried out using SPSS version 25.0. The mean and standard deviation were used to describe the continuous variables, whereas the proportion was used to describe categorical variables. Chi-square test was used for comparison between two or more groups. To analyze the relationship between dependent and independent variables, binary logistic regression and multinomial logistic regression were used. Significance was set at *p* < 0.05.

## Results

### Socio-Demographic Characteristics of Participants

In this survey, there were 12,352 males (69.9%) and 5,326 females (30.1%). Freshman to Senior year students accounted for 36.2, 32.4, 21.8, and 9.6%, respectively. Participants were mostly Han (16,634; 94.1%) and unmarried students (17,509; 99.0%). Students with monthly living expenses of 1,000–2,000 CNY accounted for the largest proportion accounted for 59.4% (Table 1).

**Table 1:** Demographic characteristics of participants.

**Variables**	***N* (%)**	**Male (*N* = 12,352)**	**Female (*N* = 5,326)**
**Grade**			
Freshman	6,392 (36.2)	4,618	1,774
Sophomore	5,740 (32.4)	4,014	1,726
Junior	3,853 (21.8)	2,529	1,324
Senior	1,693 (9.6)	1,191	502
**Nationality**			
Han	16,634 (94.1)	11,617	5,017
Minority	1,044 (5.9)	735	309
**Marital status**			
Unmarried	17,509 (99.0)	12,246	5,263
Married	169 (1.0)	106	63
**Monthly living expenses**			
≤ 1,000 CNY	6,157 (34.8)	4,577	1,580
1,000–2,000 CNY	10,496 (59.4)	7,123	3,373
2,000-3,000 CNY	782 (4.4)	490	292
≥3,000 CNY	243 (1.4)	162	81

### The Reliability and the Validity Examination

**Figure 1:**
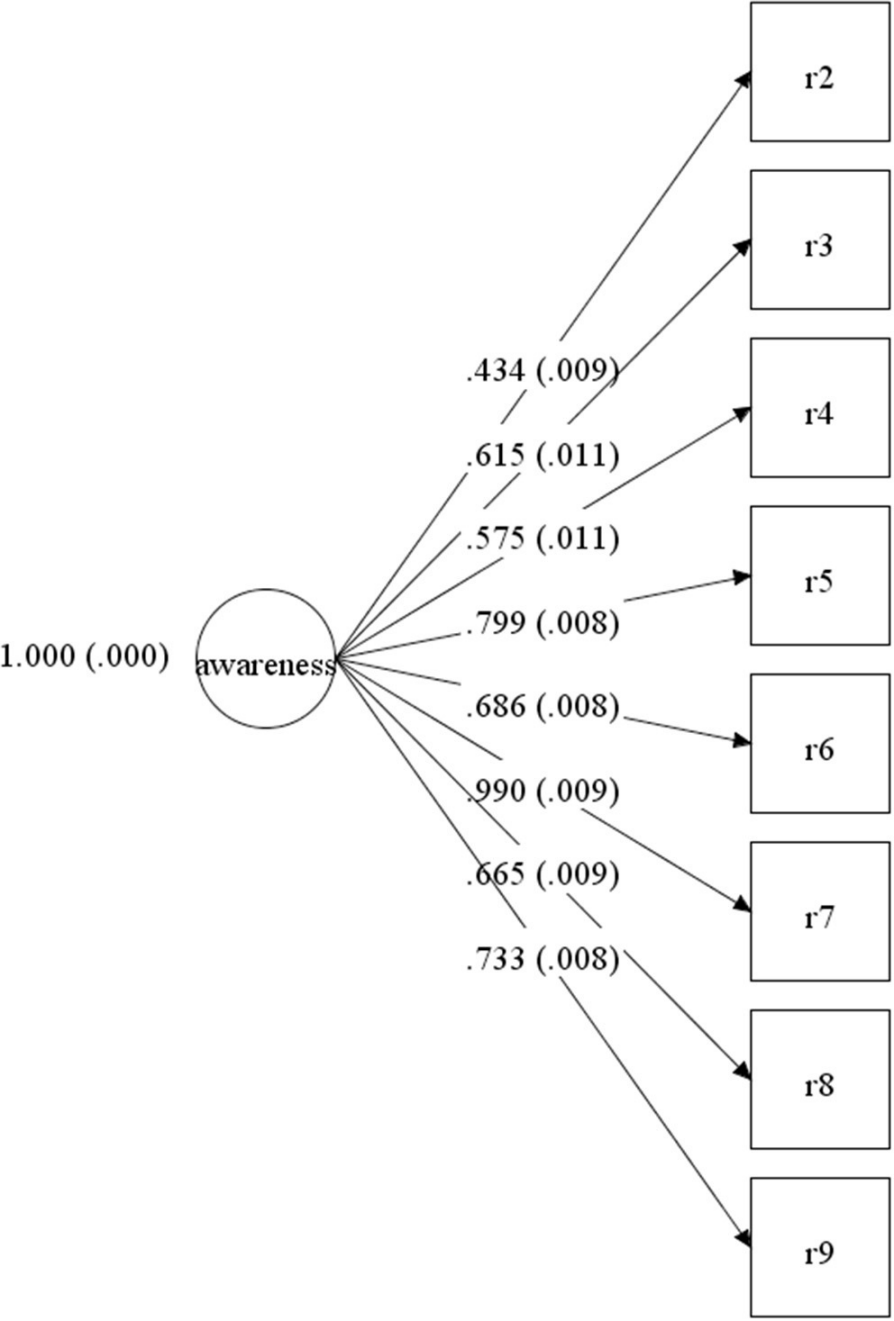
The construct validity of the questionnaire.

The reliability and validity tests of the HIV Health Education Knowledge Survey Module were conducted by internal consistency reliability, convergent validity and construct validity. The internal consistency reliability of the questionnaire was tested by calculating the Cronbach's α coefficient through SPSS 26.0. Confirmatory factor analysis (CFA) was conducted to examine the construct validity through Mplus 8.4. First, KMO and Bartlett test were performed. The KMO value of our scale was 0.845, which was higher than the threshold value of 0.6, and the Bartlett's test of sphericity reached statistical significance (*p* < 0.001), indicating that it was suitable for exploratory factor analysis (EFA). Principal-component factor models with maximum variance orthogonal rotation were used for EFA. The factor loadings are >0.4 except for the first item (0.328). We removed item 1 and conducted reliability and validity tests of the remaining 8 items. Results of CFA showed that the comparative fit index (CFI) was 0.957, Tucker-Lewis fit index (TLI) was 0.940, standardized root mean square residual (SRMR) was 0.044, root mean square error approximation (RMSEA) was 0.055 and all factor loadings were >0.40. The value of average variance extracted (AVE) was 0.882. The Cronbach's α coefficient of our scale was 0.726. Results indicated that the scale displayed good reliability and validity. Details were shown in Table 2 and Figure 1.

**Table 2:** The reliability of the questionnaire.

**Questions**	**Cronbach's α**	**Deleted entry Cronbach's α**
The incidence of AIDS among young Chinese students is increasing, and the main mode of transmission is male homosexual behavior, right?	0.729	0.733
Can it be judged by appearance that a person is infected with AIDS?		0.708
May AIDS be contracted through daily life and studying?		0.712
Can proper use of condoms reduce the risk of contracting and spreading AIDS?		0.684
Will drug abuse increase the risk of contracting AIDS?		0.696
After a high-risk behavior occurs, should we seek HIV testing and counseling?		0.689
Are the rights of HIV-infected people such as marriage/employment/schooling protected?		0.703
Is it necessary to use condoms when sex with acquaintances?		0.692

### Correct Rate of HIV Health Education Knowledge

**Figure 2:**
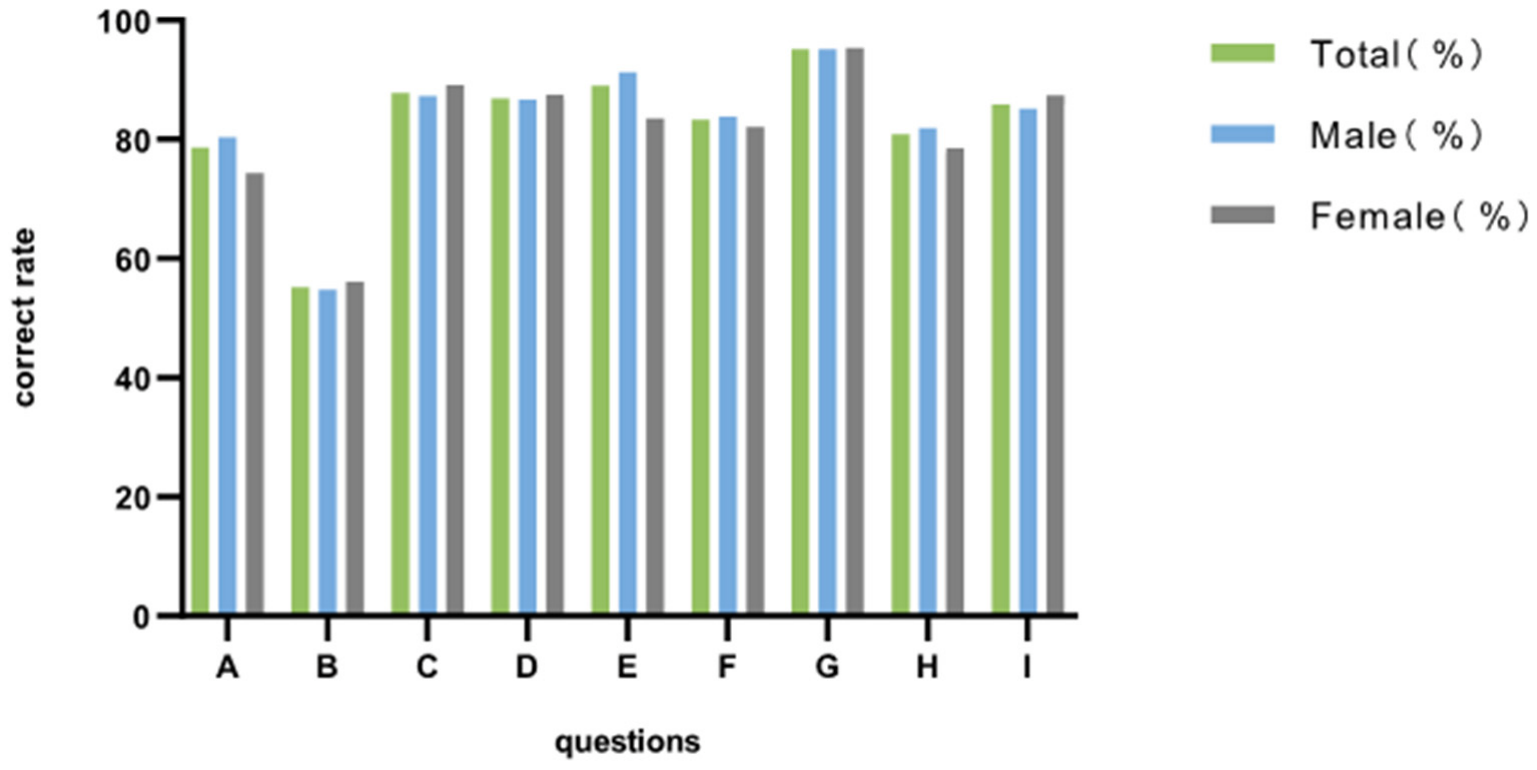
The correct rate of participants' HIV health education knowledge by gender. (A) AIDS is an incurable disease. (B) The incidence of AIDS among young Chinese students is increasing, and the main mode of.transmission is male homosexual behavior. (C) It can be judged by appearance that a person is infected with AIDS. (D) AIDS may be contracted through daily life and studying. (E) Proper use of condoms can reduce the risk of contracting and spreading AIDS. (F) Drug abuse will increase the risk of contracting AIDS. (G) After a high-risk behavior occurs, we should seek HIV testing and counseling. (H) The rights of HIV-infected people such as marriage/employment/schooling are protected. (I) Sex with acquaintances also need to use condoms.

Among the 9 questions, the one with the lowest correct rate was the mode of transmission of AIDS among Chinese adolescents, with a correct rate of 55.2%. The question with the highest correct rate is seeking HIV testing and counseling after high-risk behaviors, with a correct rate of 95.2%. There were 15,370 people (86.9%) who know that they would not be infected by exposure to AIDS in daily life. Generally speaking, the correct rate of each question was about the same between boys and girls (Figure 2).

### HIV Awareness and Knowledge

The overall HIV health education knowledge awareness rate of the survey respondents was 80.8%, and the average score of the HIV health education knowledge part was 7.93 (±1.64). Among them, 4,679 people answered all 9 questions correctly, accounting for 26.5% of the total survey respondents.

**Figure 3:**
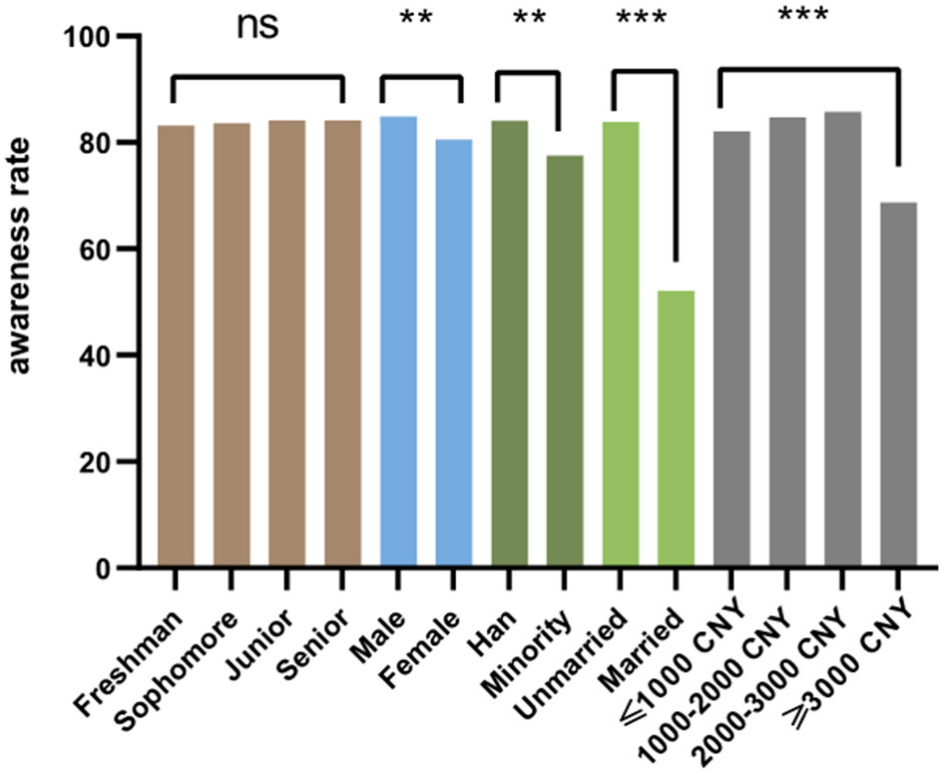
Awareness rate of AIDS-related knowledge among college students. ns *p* > 0.05, *p < 0.05, **p < 0.01, ***p < 0.001.

**Figure 4:**
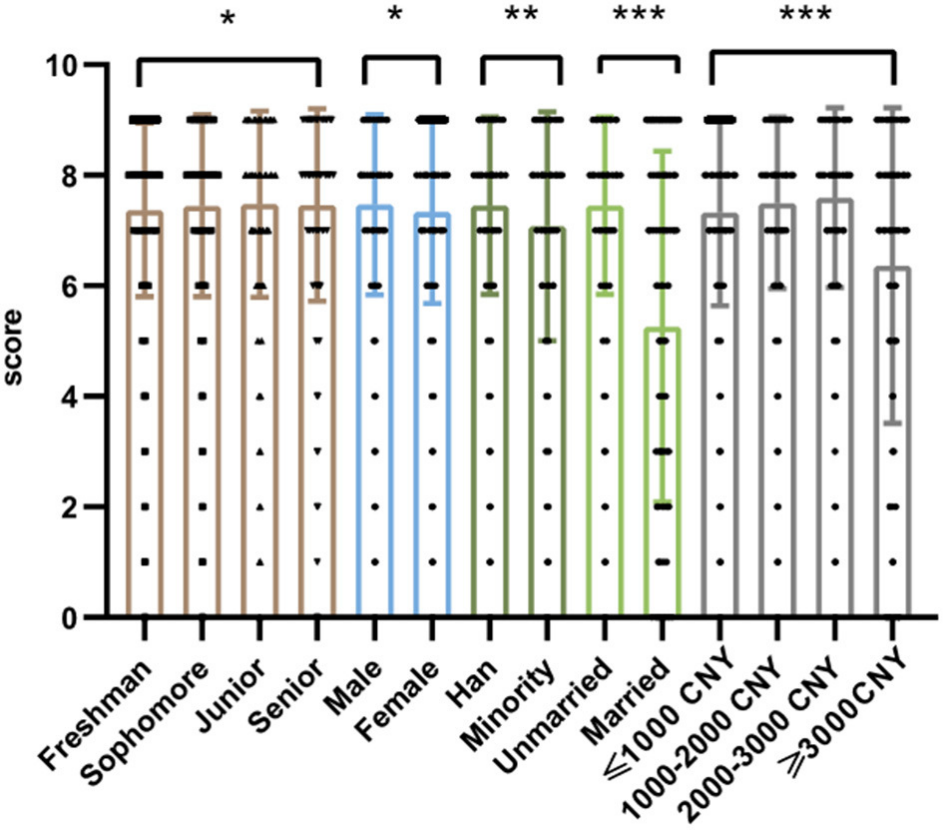
Score of AIDS-related knowledge among college students. ns *p* > 0.05, *p < 0.05, **p < 0.01, ***p < 0.001.

Calculated the proportions of awareness of different demographic characteristics, and compared them with the Chi-square statistical test. It can be known that the third-year students in the grade have the best awareness, boys have better awareness than girls, Han students have better awareness than minority students, unmarried students have better cognition than unmarried students, and participants whose living expenses are in the range of 2,000–3,000 CNY have the best awareness. There were differences in the knowledge of AIDS health education among college students of different grades, genders, ethnicities, marital status, and living expenses, and the differences were statistically significant (*p* < 0.05) (Figures 3, 4).

### Influencing Factors of Knowledge About AIDS Health Education

The results of multivariate logistic regression showed that the participants' awareness were associated with their gender, ethnicity, and marital status (*p* < 0.05). Specifically, boys were 1.268 times more likely to be aware of reaching the standard than boys (OR = 1.268, 95% CI = 1.162–1.384); among participants from different nationalities, Han students were 1.4 times more likely than ethnic minority students to meet the standard (OR = 1.400, 95% CI = 1.194–1.641); unmarried students were more than 3 times more likely to meet the standard than married students (OR = 3.957, 95% CI = 2.846–5.502) (Table 3).

**Table 3:** Multivariate unconditional logistic regression on awareness of HIV health education knowledge.

**Variables**	***B***	***P***	**OR**	**95% CI**
**Gender**				
Male	0.237	<0.001	1.268	1.162–1.384
Female (Ref.)				
**Nationality**				
Han	0.337	<0.001	1.400	1.194–1.641
Minority (Ref.)				
**Marital status**				
Unmarried	1.375	<0.001	3.957	2.846–5.502
Married (Ref.)				
**Monthly living expenses**				
≤ 1,000 CNY	0.301	0.059	1.352	0.989–1.847
1,000–2,000 CNY	0.594	<0.001	1.812	1.329–2.471
2,000–3,000 CNY	0.639	0.001	1.895	1.315–2.732
≥3,000 CNY (Ref.)				

### Attitudes Toward Live With HIV-Positive Roommates

In this survey, in the question “Are you willing to share a dormitory with a roommate who is infected with HIV/AIDS?” 3,667 people chose “Yes,” accounting for 20.7%; 9,201 people chose “unwilling,” accounting for 52.0%; 4,810 people chose “uncertain,” accounting for 27.2%. Among them, those who choose “Yes” were considered to have a positive attitude toward AIDS patients, while at the same time, those who choose “No” were considered to have a negative attitude.

**Figure 5:**
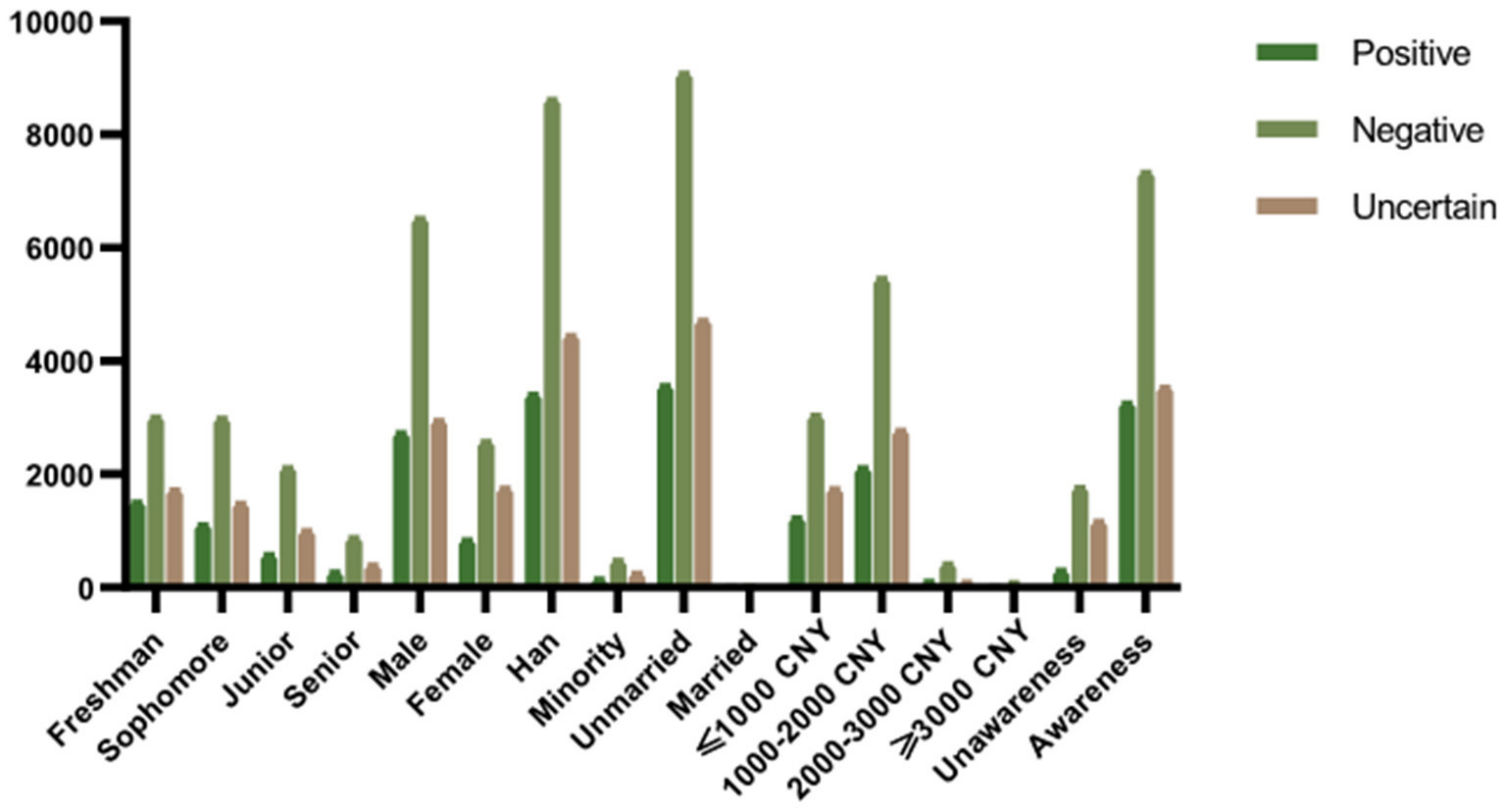
Attitudes towards live with HIV-positive roommates.

The results indicated that freshmen students had shown to be more accepting of living with a HIV-positive roommate. More boys than girls among participants who had a positive attitude toward AIDS patients. Moreover, among participants with different monthly living expenses, those with more than 3,000 CNY had the most positive attitudes. Additionally, People who had a better understanding of HIV/AIDS-related knowledge accept AIDS patients better. The above differences are statistically significant (*p* < 0.01) (Figures 4, 5).

### Influencing Factors of Attitudes Toward Live With HIV-Positive Roommates

Taking different demographic characteristics and awareness as independent variables, whether they are willing to share a dormitory with students who are infected with HIV/AIDS (1 = yes; 2 = no; 3 = uncertain) as dependent variables, taking the last item in each category as the control group. Multivariate logistic regression were performed. The analysis showed that the gender, marital status and knowledge of college students had varying degrees of influence on their attitudes to live with HIV-positive roommates:

“Positive” compared to “negative.” Male students were 0.845 times more likely to have a negative attitude than female students (OR = 0.845, 95% CI = 0.773–0.924); The possibility that those who did not meet the standard were unwilling to live with HIV-infected patients was 2.753 times that of those who met the standard (OR = 2.753, 95% CI = 2.391–3.170).

“Positive” compared to “uncertain.” Male students were 0.552 times more likely to choose “uncertain” than female students (OR = 0.552, 95% CI = 0.501–0.609). Compared with those who met the standard of AIDS health education knowledge, those who did not meet the standard are 3.569 times more likely to choose “uncertain” (OR = 3.569, 95% CI = 3.079–4.137) (Table 4).

**Table 4:** Multivariate logistic regression of the attitudes to live with HIV positive roommates.

**Variables**	**Negative (%)**				**Uncertain (%)**			
	**B**	***P***	**OR**	**95% CI**	**B**	***P***	**OR**	**95% CI**
**Grade**
Freshman	−0.427	<0.001	0.652	0.566–0.752	−0.227	0.006	0.797	0.678–0.937
Sophomore	−0.123	0.095	0.884	0.765–1.022	−0.063	0.455	0.939	0.796–1.108
Junior	0.167	0.036	1.181	1.011–1.381	0.162	0.073	1.176	0.985–1.404
Senior (Ref.)								
**Gender**
Male	−0.168	<0.001	0.845	0.773–0.924	−0.594	<0.001	0.552	0.501–0.609
Female (Ref.)								
**Nationality**
Han	−0.037	0.669	0.964	0.814–1.142	−0.150	0.117	0.861	0.714–1.038
Minority (Ref.)								
**Marital status**
Unmarried	0.922	<0.001	2.514	1.715–3.686	0.737	0.001	2.089	1.342–3.252
Married (Ref.)								
**Monthly living expenses**
≤ 1,000 CNY	−0.008	0.963	0.992	0.718–1.372	0.838	<0.001	2.311	1.505–3.548
1,000–2,000 CNY	0.089	0.588	1.093	0.793–1.507	0.780	<0.001	2.182	1.425–3.341
2,000–3,000 CNY	0.220	0.238	1.246	0.865–1.794	0.381	0.119	1.463	0.907–2.360
≥3,000 CNY (Ref.)								
**Awareness of HIV/AIDS**
Unawareness	1.013	<0.001	2.753	2.391–3.170	1.272	<0.001	3.569	3.079–4.137
Awareness (Ref.)								

## Discussion

This study aims to describe the current situation of HIV health education among Chinese college students and the acceptance of contemporary college students to live in the same room with HIV-positive roommates, then analyze its influencing factors. The results of this study showed that the overall awareness rate of HIV health education knowledge of the university is 80.8%. Compared with previous studies in this province, it is lower than the HIV knowledge awareness rate (84.7%) of college students in Henan Province in the study of Yin et al. (18) in 2017. Compared with other provinces, the overall awareness rate is higher than that of Jining college students in the study by Liu et al. (19) (66.59%), but lower than that of Chen et al. (20) in the study of Central Nantong University (87.87%). The different awareness of various local universities may be related to the way and intensity of HIV prevention publicity and education work in different regions and schools. Our survey found that only 26.5% of the participants correctly answered all 9 questions about HIV health education knowledge. The lowest correct rate is “At present, the prevalence of HIV among young students in China is increasing rapidly. The main mode of transmission is male homosexual sex, followed by heterosexual sex.” Only 55.2% answered correctly. It shows that the students of this school do not fully understand the main transmission mode of HIV among the young population and the risks of unprotected sex. This result is similar to the results of several foreign studies (6, 7, 21); the highest accuracy rate is “After the occurrence of high-risk behaviors (sharing needles, drug use, unsafe sex, etc.), you should actively seek HIV testing and counseling” accounted for 95.2% of the total answers. This result shows that students have a strong sense of self-protection after high-risk sexual behaviors occur. The difference in the accuracy of these two questions also shows from the side that college students' learning of HIV health education knowledge is not systematic and complete, and the accuracy of general problems is always higher, but the accuracy of some professional problems is obviously lower (22).

In terms of humanity characteristics and HIV health education knowledge, we found that the awareness of boys is better than that of girls. This is the same as the results of some previous studies (22–24). This is understandable, as girls are likely to have a more conservative attitude toward sexual behavior than boys (23, 25). There is not much difference between college students with different living standards, but overall, there is a trend that the higher the living expenses level, the higher the awareness rate. On one hand, for college students in the same university, it may be because they come from regions with different economic levels. Students from regions with high economic levels are more likely to have earlier and more comprehensive exposure to sex education, so they have a better understanding of HIV/AIDS-related knowledge (26). On the other hand, it may also be because their family pays more attention to sex education in the family. This suggests that we should pay attention to differences caused by different factors such as gender and region, and carry out targeted HIV health education. Schools should regularly hold AIDS health education lectures or relevant theoretical courses. Some studies have found that peer education has a greater advantage in sex education because their growth backgrounds are similar and easy to communicate with (20). It is recommended that colleges and universities try to let medical students assist schools to carry out regular health counseling activities after training. At the same time, give full play to the role of clubs and other platforms in schools, encourage students to actively participate in AIDS publicity and education activities, and further strengthen AIDS publicity and education in colleges and universities.

Regarding the relationship between HIV health education knowledge level and attitudes to live with HIV-positive roommate. The analysis of our study found that people with AIDS awareness standards were more likely to accept cohabitation with HIV-positive roommates than people who did not meet the standards. This is consistent with the conclusion in other literature that people with better HIV/AIDS-related knowledge awareness have a more positive attitude toward HIV-infected patients (27). The development of publicity and education on HIV prevention among college students will not only help college students prevent HIV, but also help eliminate discrimination against HIV-infected patients. However, although 86.9% of students know that they will not be infected with HIV in daily life and study contact, 52% of students still do not want to live with HIV-positive roommates. This result shows that even if you have a certain understanding of HIV/AIDS-related knowledge, it is still difficult to accept emotionally. This suggests that psychological prevention and treatment should also be paid attention to during HIV health education.

Due to the influence of traditional culture, Chinese people's attitudes toward “sex” has always been relatively conservative, At the same time, comprehensive sex education has not been widely and comprehensively carried out in China., sexual education is very lacking in both school education and family education (10, 28, 29). Someone compared Chinese and foreign sex education policies in 2019. The initial education time for sex education in China started in junior high school, much later than the internationally stipulated 5–8 years old (30). In addition to the lack of middle school education, many universities in China are not offering sexual education and do not have sound HIV test facilities (31).

Relevant studies around the world have shown that educational intervention can effectively improve people's HIV health education knowledge, effectively increase student population's awareness of HIV/AIDS-related knowledge (5) and willingness to test for HIV (32), and improve people's discrimination against AIDS groups (33). In today's internet age, schools can adopt a variety of new ways to educate students on AIDS prevention and treatment, such as the PBL method that integrates clinical actual cases into teaching materials (34), the theater-based AMP method (35), and the GBL method based on games (36). Compared with traditional book-based teaching, these new educational methods integrate knowledge into real life and present it to people in a more vivid way. It is easier to arouse the interest of young people and thus obtain better educational effects.

### Limitations

The limitation of this study is that this survey was conducted only in one university, which is somewhat different from the overall situation of the country. In addition, this study did not involve factors such as the participants' majors, hometown, parents' marital status, which may also affect their level of HIV/AIDS-related knowledge. Despite the limitations referred to above, the results of this study can still provide references for the communication of HIV health education knowledge among college students nationwide.

## Conclusions

The results of this study showed that this college students had a certain understanding of HIV health education knowledge, but they did not know enough about the sexual transmission of HIV. Meanwhile, many students showed a relatively negative attitude toward HIV-infected groups. What needs attention is the cognition of AIDS among female students, they showed a greater lack of awareness of AIDS in this study. Therefore, in the future HIV/AIDS-related work, it is necessary to strengthen the education of women. The results provided by this study showed that raising awareness of AIDS health education knowledge can effectively alleviate people's negative attitudes toward HIV-infected patients. This suggests that China should carry out targeted sex education and psychological education as soon as possible, and pay attention to gender characteristics in the popularization of HIV/AIDS-related knowledge. Furthermore, education methods can learn from some new foreign education methods to improve students' awareness of HIV/AIDS-related knowledge more efficiently.

## Data Availability Statement

The raw data supporting the conclusions of this article will be made available by the authors, without undue reservation.

## Ethics Statement

The studies involving human participants were reviewed and approved by the Ethics Committee of Wuhan University approved this study (Approval ID: 2021YF0047). The patients/participants provided their written informed consent to participate in this study.

## Author Contributions

LZ designed the survey, analyzed the data, and reviewed manuscript. HY analyzed the data, wrote the manuscript, and prepared figures and tables. HL designed the survey and collected the data. WR, XM, and XD analyzed the data and wrote the manuscript. XT edited the manuscript. All authors contributed to the article and approved the submitted version.

## Conflict of Interest

The authors declare that the research was conducted in the absence of any commercial or financial relationships that could be construed as a potential conflict of interest.

## Publisher's Note

All claims expressed in this article are solely those of the authors and do not necessarily represent those of their affiliated organizations, or those of the publisher, the editors and the reviewers. Any product that may be evaluated in this article, or claim that may be made by its manufacturer, is not guaranteed or endorsed by the publisher.

## Abbreviations

HIV: Human Immunodeficiency Virus
AIDS: Acquired Immunodeficiency Syndrome
OR: Odds Ratio
CI: Confidence of Interval
CDC: Chinese Center for Disease Control and Prevention
CNY: Chinese yuan
PBL: Problem-Based Learning
AMP: Arts-based, Multiple component, Peer-education
GBL: Game-Based Learning
SPSS: Statistical Product and Service Solutions.
